# Genomic and transcriptomic analyses reveal a Gifsy-1 prophage-encoded virulence module associated with enhanced epithelial adhesion of *Salmonella* ST34 in Huzhou, China

**DOI:** 10.3389/fcimb.2026.1849969

**Published:** 2026-07-24

**Authors:** Ling Zhu, Xiangchen Li, Lili Zheng, Lihua Zhou, Zhenfa Chen, Yibin Lin, Jianhua Zhu, Junshun Gao, Junli Gao, Yunxiang Cai, Xinhua Qiang

**Affiliations:** 1 Department of Clinical Laboratory, First Affiliated Hospital of Huzhou Normal University, The First People's Hospital of Huzhou, Huzhou, China; 2 Cosmos Wisdom Mass Spectrometry Center of Zhejiang University Medical School, Hangzhou, China; 3 School of Medicine, Huzhou Normal University, Huzhou, China

**Keywords:** antimicrobial resistance, comparative genomics, Gifsy-1 prophage, *Salmonella*, ST34, transcriptomics, virulence

## Abstract

**Introduction:**

Non-typhoidal *Salmonella* (NTS) is a leading cause of bacterial gastroenteritis worldwide, yet the pathogenic differences among co-circulating dominant clones remain poorly understood.

**Methods:**

A 7.5-year surveillance study (2017–2024) was conducted using NTS isolates from patients with acute diarrheal illness in Huzhou, China. Quantitative IEC-6 cell-based adhesion and invasion assays, comparative genomics, transcriptomic profiling, and protein-protein interaction network analysis were integrated to compare the dominant sequence types (STs).

**Results:**

Among 188 NTS isolates, 35 serotypes and 36 STs were identified. Three STs predominated: ST34 (26.1%, mainly monophasic *S. Typhimurium*), ST11 (15.4%, exclusively *S. Enteritidis*), and ST19 (13.3%, classical *S. Typhimurium*). ST34 showed significantly enhanced epithelial adhesion in IEC-6 cells, whereas increased invasion was observed only in comparison with ST11 and not with ST19. Comparative genomics and transcriptomics identified an intact Gifsy-1 prophage as an ST34-specific genetic element encoding the T3SS effectors GogB and SteE/SarA, with *gogB* and *steE/sarA* being the only virulence genes consistently upregulated in ST34 versus both ST11 and ST19. Protein-protein interaction network analysis positioned these effectors as peripheral components of a 14-member prophage-associated module. All 49 ST34 isolates carried the intact Gifsy-1 prophage, whereas ST19 isolates harbored only truncated versions and ST11 completely lacked this element. ST34 also carried a broad antimicrobial resistance gene repertoire, including frequent tetracycline and sulfonamide resistance determinants and sporadic detection of clinically important beta-lactamase genes.

**Discussion:**

These findings identify an intact Gifsy-1 prophage encoding GogB and SteE/SarA as a candidate module associated with enhanced epithelial adhesion in ST34, a dominant NTS lineage with an extensive antimicrobial resistance repertoire.

## Introduction

Non-typhoidal *Salmonella* (NTS) represents one of the leading causes of bacterial foodborne gastroenteritis worldwide, causing an estimated 93 million enteric infections and 155,000 deaths annually (Majowicz et al., 2010). While most NTS infections manifest as self-limiting acute diarrheal illness, severe invasive forms can occur, particularly in young children, elderly, and immunocompromised individuals, leading to substantial morbidity and healthcare burden (Kumar et al., 2025). The clinical outcomes of NTS infection are largely determined by the complex interplay between host immune status and bacterial virulence potential, with certain *Salmonella* serotypes exhibiting enhanced capacity to cause invasive disease (Gilchrist et al., 2015).

*Salmonella enterica* subspecies *enterica* encompasses more than 2,600 serotypes, among which *S. Typhimurium* and *S. Enteritidis* have consistently ranked as the most prevalent serotypes associated with human infections globally (Mezal et al., 2014). However, the application of multilocus sequence typing (MLST) has revealed substantial genetic heterogeneity within these serotypes, with distinct sequence types (STs) exhibiting differential geographic distribution, host adaptation, and clinical phenotypes (Achtman et al., 2012). In recent decades, ST34 has emerged as a dominant clone of monophasic *S. Typhimurium* across Asia and Europe, particularly associated with swine production chains and showing alarming rates of multidrug resistance (Pardos de la Gandara et al., 2023; Wang et al., 2023b). Concurrently, ST11 (exclusively corresponding to *S. Enteritidis*) and ST19 (classical *S. Typhimurium*) remain persistently prevalent in both human and animal reservoirs (Yan et al., 2021; Wang et al., 2023b). Despite their co-circulation, systematic comparisons of the pathogenic potential and underlying molecular mechanisms among these three dominant STs remain surprisingly limited (Wu et al., 2025).

China bears a substantial burden of NTS infections, with surveillance studies reporting increasing isolation rates and alarming expansion of antimicrobial resistance (Wang et al., 2023b). Nevertheless, most studies have focused on serotype-level surveillance, and comprehensive genomic characterization of dominant clones at the regional level remains scarce (Wang et al., 2023b; Wu et al., 2025). Furthermore, while genomic epidemiology has successfully traced the dissemination of successful clones, the functional consequences of genetic differences—particularly those related to virulence potential—have rarely been experimentally validated (Wang et al., 2023a). Understanding whether predominant STs differ in their intrinsic pathogenic capacity and identifying the genetic basis underlying such differences is critical for risk assessment and the development of targeted intervention strategies.

In this study, we conducted a comprehensive 7.5-year surveillance of NTS isolates from patients with acute diarrheal illness in Huzhou, China, revealing the striking predominance of three sequence types: ST34, ST11, and ST19. Through integrated analyses combining comparative genomics, quantitative cell-based infection assays, and transcriptomic profiling, we show that ST34 exhibits significantly enhanced epithelial adhesion in IEC-6 cells, whereas increased invasion is observed only in comparison with ST11 and not with ST19. Comparative genomic and transcriptomic analyses identified an intact Gifsy-1 prophage region encoding the T3SS effectors *gogB* and *steE*/*sarA* as a ST34-specific candidate virulence -associated module. Our findings identify a prophage-encoded candidate virulence module associated with the enhanced epithelial adhesion phenotype of the emerging ST34 clone.

## Materials and methods

### Isolation and identification of *Salmonella* from acute diarrheal cases

Fecal samples from patients with acute infectious diarrhea, collected at our hospital from January 2017 to June 2024, were streaked onto Columbia blood agar, CHROMagar Orientation, SS agar, and Vibrio chromogenic agar, then incubated at 37 °C. Samples were also enriched in alkaline peptone water for 6–8 hours and subcultured on CHROMagar Orientation. Suspicious colonies were identified using MALDI-TOF MS (EXS3000) by smearing single colonies onto a target plate, treating with 1 μL formic acid and 1 μL matrix solution, air-drying, and analyzing. *Salmonella* isolates were serotyped in a BSL-2 cabinet using a commercial antisera agglutination kit, mixing bacterial suspensions with polyvalent and monovalent O and H antisera on a glass slide to determine serotypes based on agglutination patterns. Confirmed isolates were stored in 30% glycerol broth at –80 °C.

### Antimicrobial susceptibility testing

Antimicrobial susceptibility testing of the NTS isolates was performed against 12 antibiotics—ampicillin (AMP), ampicillin/sulbactam (AMC), aztreonam (ATM), cefepime (FEP), ceftazidime (CAZ), ceftriaxone (CRO), ciprofloxacin (CIP), levofloxacin (LEV), imipenem (IPM), ertapenem (ETP), piperacillin/tazobactam (TZP), and trimethoprim/sulfamethoxazole (SXT)—using the VITEK 2 Compact system with GN13 cards. Following the CLSI M100 (33rd edition) breakpoints, a fresh colony was suspended in 0.45% saline, adjusted to 0.5 McFarland standard, and 145 µL of the suspension was transferred for card inoculation; MICs were read after overnight incubation, with *Escherichia coli* ATCC 25922 serving as the quality control strain.

### *Salmonella* adhesion and invasion assay​

A total of 102 frozen NTS isolates (ST34, ST19 and ST11) were revived on Columbia blood agar (37 °C, 18–24 h), and single colonies were cultured in LB broth (37 °C, 150 rpm, 6 h) before adjusting the bacterial density to OD600 ≈0.3 (~10^8^ CFU/mL). The IEC-6 cell line (obtained from Saiku Biological Technology Co., Ltd., Guangzhou, China) was employed as a reproducible intestinal epithelial model, consistent with its established use in studying *Salmonella Typhimurium* invasion and associated host responses (Chaussé et al., 2023). Cells were thawed, resuspended in DMEM with 1% penicillin-streptomycin, centrifuged (1000 rpm, 5 min), and cultured in T25 flasks (37 °C, 5% CO_2_). Upon reaching confluence, cells were trypsinized (0.25% EDTA), neutralized with 10% FBS-DMEM, and passaged three times for stabilization. For adhesion assays, cells were digested, resuspended in antibiotic-containing DMEM, adjusted to 10^4^ cells/mL, and seeded into 24-well plates. At 80–90% confluence, the medium was replaced with antibiotic-free DMEM for subsequent bacterial adhesion and invasion testing.

Based on preliminary optimization of the relationship between OD600 and bacterial counts, *Salmonella* strains were cultured under the same conditions and adjusted to OD600 ≈ 0.3, corresponding to approximately 10^8^ CFU/mL. The initial inoculum was further quantified by CFU plating. Bacterial suspensions were serially diluted (10⁻^6^ to 10⁻^7^) in PBS, plated on LB agar (50 μL/plate), and incubated (37 °C, 5% CO_2_, 18–24 h) to determine initial CFU counts. For adhesion and invasion assays, *Salmonella* strains were adjusted to an MOI of 100:1 (bacteria:IEC-6 cells) and added to 24-well plates in triplicate wells per strain, with DMEM-only wells included as negative controls. Adhesion and invasion values for each strain were calculated as the mean of the three technical replicate wells. Plates were centrifuged (1000 rpm, 10 min) and incubated (37 °C, 5% CO_2_, 90 min). For adhesion assessment, wells were either fixed with 4% PFA, Wright-Giemsa stained with solutions A/B, and washed with PBS, or lysed with 0.5% Triton X-100 at 37 °C for 30 min. Lysates were diluted (10⁻¹ to 10⁻³) and plated for CFU enumeration. Invasion assays followed the same procedure after gentamicin treatment (50 μg/mL, 1 h), as previously described in *Salmonella* invasion assays, to kill extracellular bacteria (Liu et al., 2024). Adhesion and invasion rates were expressed as per mille (‰) and calculated as (adhered or invaded CFU/initial CFU) × 1000. Statistical analyses were performed using R software (version 4.1).

### WGS and genomics analysis

Genomic DNA of *Salmonella* spp. was extracted using either the SDS or STE method. DNA purity and integrity were assessed by agarose gel electrophoresis, and concentration was measured using a Qubit fluorometer. High-quality DNA samples were randomly sheared to ~350 bp fragments using a Covaris ultrasonicator. Library preparation was performed with the NEBNext^®^ Ultra™ DNA Library Prep Kit for Illumina (NEB, USA), following standard steps including end repair, A-tailing, adapter ligation, purification, and PCR amplification. The resulting libraries were first quantified using Qubit 2.0 and diluted to 2 ng/μL. Insert sizes were assessed with the Agilent 2100 Bioanalyzer. Libraries with expected insert sizes were further quantified using qPCR to ensure effective concentration and quality. WGS was carried out on the Illumina NovaSeq 6000 platform with paired-end 150 bp reads (PE150).

Raw reads were quality-trimmed using fastp v0.23.2 and assembled with Unicycler v0.5.0 using default parameters (Wick et al., 2017; Chen et al., 2018). Gene prediction and functional annotation were performed with Bakta v1.11.0 (Schwengers et al., 2021). Sequence types (STs) were determined by submitting assemblies to the PubMLST database (Jolley et al., 2018). Salmonella serotypes were identified by SeqSero2 v1.3.1 (Zhang et al., 2019). Acquired antimicrobial resistance (AMR) genes and mutations were identified using the Resfinder database, while virulence factors (VFs) were screened against the VFDB database (Florensa et al., 2022; Liu et al., 2022). The core genome alignment and SNP calling (cgSNP) were conducted using Parsnp v1.2 from the HarvestTools suite (Treangen et al., 2014). Recombination regions were filtered out with Gubbins v3.3 (Croucher et al., 2015). Maximum likelihood phylogeny was reconstructed with IQ-TREE2 using the HKY model, and branch support was evaluated with 1000 ultrafast bootstrap replicates (Nguyen et al., 2015). The resulting phylogenetic tree was visualized using the Interactive Tree of Life (iTOL) web tool (Letunic and Bork, 2021).

The pangenome was reconstructed with Panaroo v1.5.0 in “moderate” mode under default settings (Tonkin-Hill et al., 2020). The resulting gene presence-absence matrix, together with associated metadata, was analyzed in R using the Pagoo framework for principal component analysis (PCA) and for delineating pangenome structure (Ferrés and Iraola, 2021). Pangenome size and openness were estimated with the micropan package according to Heap’s law, and genomic fluidity was also calculated (Snipen and Liland, 2015). Gene gain and loss rates between subspecies were derived using Panstripe v0.3 (Tonkin-Hill et al., 2023).

### RNA-seq and bioinformatics analysis

Three strains each of ST11, ST19, and ST34 were randomly selected, yielding nine samples for transcriptomic analysis. These strains were grown in LB broth at 37 °C with shaking (150 rpm) to mid-log phase (OD600 ≈ 0.6), and RNA was stabilized using RNAprotect^®^ Bacterial Reagent (Qiagen). Total RNA was extracted with the RNeasy Mini Kit (Qiagen) including on-column DNase I treatment, and quality was assessed using a NanoDrop spectrophotometer and Agilent Bioanalyzer (RIN ≥ 8.0). Ribosomal RNA was removed with the Ribo-Zero™ rRNA Removal Kit (Illumina), and strand-specific cDNA libraries were prepared using the NEBNext^®^ Ultra™ II RNA Library Prep Kit (NEB). Libraries were quality-checked on an Agilent Bioanalyzer and quantified via qPCR (KAPA Biosystems). Paired-end sequencing (PE150) was performed on an Illumina NovaSeq 6000, yielding approximately 20 million reads per sample.

Raw reads were quality-checked and trimmed using fastp v0.26 to remove adapters and low-quality bases (Phred < 20) (Chen et al., 2018). Clean reads were aligned to the reference genome of the ST34 *Salmonella* strain A60, as determined by WGS in this study, using HISAT2 software (Kim et al., 2019). For RNA-seq quality assessment, we summarized the total reads, genome-mapped reads, unique mapping rates, and rRNA contamination rates for each sample. Sequencing saturation and gene-body coverage distribution curves were also generated to evaluate whether sequencing depth was sufficient and whether reads were evenly distributed across gene bodies. Subsequently, transcript assembly and expression quantification were performed with StringTie v2.2.3 (Pertea et al., 2015), followed by differential expression analysis using DESeq2 v1.38.0 (Love et al., 2014). Pearson correlation analysis was performed using R (version 4.1). The coding regions (ORFs) of each transcript were predicted using TransDecoder (https://github.com/TransDecoder/TransDecoder), with only the longest ORF being reported for each transcript. Protein-protein interaction (PPI) networks for DEGs and ST-specific VFs were constructed by using STRING (v12.0) database (Szklarczyk et al., 2017).

## Results

### Predominant ST34, ST11 and ST19 of NTS in Huzhou, China

**Figure 1:**
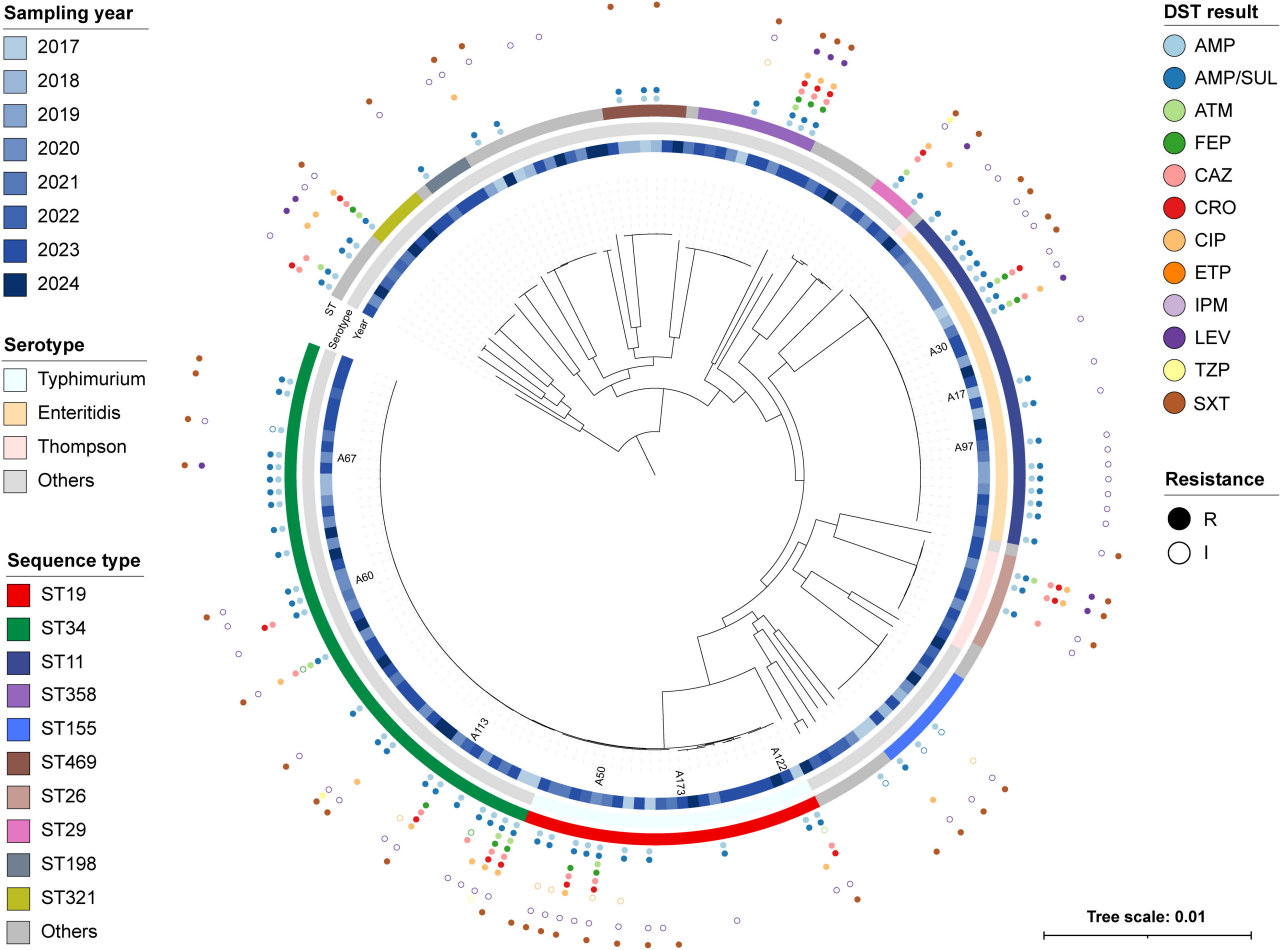
Phylogeny, genotype and resistance profile of 188 NTS strains. The phylogenetic tree was constructed from cgSNPs using the ML method, rooted at the midpoint, with branch lengths indicating the number of nucleotide substitutions per site. Concentric rings from inner to outer display sample collection time, serotype, and ST in distinct colors, while the outermost ring represents phenotypic antimicrobial susceptibility testing results for 12 antimicrobial agents, where solid circles denote resistance (R), hollow circles indicate intermediate resistance (I), and blank areas signify susceptibility (S). Three strains from each of the three major subtypes (ST11, ST19, and ST34), labeled in red, were selected for subsequent transcriptomic experiments.

From January 2017 to June 2024, 188 NTS isolates were collected from patients with acute infectious diarrhea at a tertiary hospital in Huzhou, China (**Supplementary Table 1**). All 188 isolates were included in serotyping, MLST, WGS, and antimicrobial susceptibility testing. Among these isolates, 35 distinct serotypes were identified, with *S. Typhimurium* being the most prevalent (73/188, 38.8%), followed by *S. Enteritidis* (29/188, 15.4%) (**Figure 1**). MLST analysis identified 36 distinct STs, with ST34 (49/188, 26.1%), ST11 (29/188, 15.4%), and ST19 (25/188, 13.3%) representing the predominant types. Among the 49 ST34 isolates, most were monophasic *S. Typhimurium* (47/49, 95.9%), while one was classical *S. Typhimurium* and one was *S. Essen*. Conversely, all monophasic *S. Typhimurium* isolates belonged to ST34 (47/47, 100%), classical *S. Typhimurium* isolates predominantly belonged to ST19 (25/26, 96.2%), and all *S. Enteritidis* isolates corresponded to ST11 (29/29, 100%).

Distinct STs formed separate clades on the phylogenetic tree, indicating divergent evolutionary lineages (**Figure 1**). In contrast, isolates within the same ST—such as ST34, ST19, ST11, ST40, and ST155—exhibited relatively low genetic variation. Notably, ST34, ST19, and ST11 each formed well-defined clusters, with average intra-cluster SNP differences of 49, 600, and 72, respectively. Among them, the genetic distance between ST34 and ST19 was markedly smaller than that observed between other STs. These three predominant STs were detected consistently across all sampling years from 2017 to 2024.

NTS isolates exhibited high resistance rates to AMP (70.7%, 133/188) and AMP/SUL (66.5%, 125/188) (**Supplementary Table 2**). Resistance to CAZ and CRO was observed in 17.0% (32/188) and 23.9% (45/188) of isolates, respectively. Resistance rates to CIP and LEV were 17.6% (33/188) and 10.6% (20/188), respectively. Notably, ST34 strains showed higher resistance rates to several beta-lactams and beta-lactam/beta-lactamase inhibitor combinations, including AMP, AMP/SUL, CRO, CAZ, FEP, ETP, IPM, and TZP, compared with ST19 and ST11 strains. Furthermore, two NTS isolates were resistant to both carbapenems tested, ETP and IPM. The prevalence of multidrug-resistant (MDR) strains was 31.9% (60/188).

### ST34 shows enhanced adhesion, with significantly higher invasion only compared with ST11

**Figure 2:**
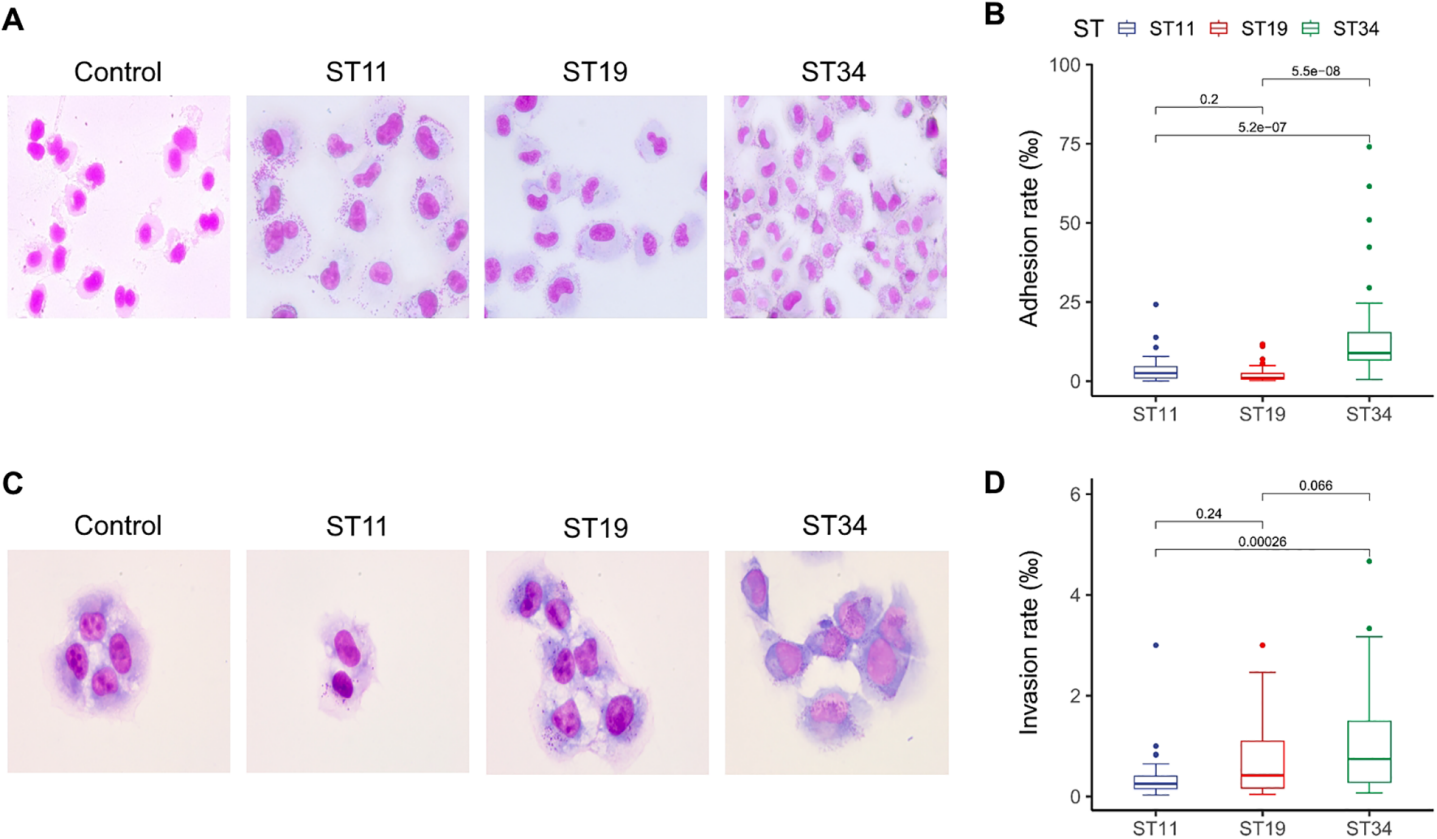
*In vitro* Cell Invasion and Adhesion Assays for ST11, ST19 and ST34 strains. **(A)** Representative micrographs showing adhesion **(A)** and invasion **(C)** of three NTS sequence types (ST11, ST19, ST34) in IEC-6 cells, with uninfected controls (leftmost panels). Corresponding box plots **(B, D)** quantify adhesion and invasion rates, respectively; pairwise differences were assessed by Wilcoxon rank-sum test (p-values shown above).

Among the three dominant STs, 102 viable isolates were included in the adhesion and invasion assays, comprising 48 ST34, 25 ST19, and 29 ST11 isolates (**Supplementary Table 3**). Although 49 ST34 isolates were included in the surveillance and genomic analyses, one ST34 isolate (A189) failed to recover from frozen storage and was therefore excluded from the cell-based assays. In the adhesion assays, the 48 isolates of ST34 exhibited a broad range of adhesion rates, from 0.59‰ to 74.00‰, with a median value of 7.91‰ (**Figures 2A, B**). In contrast, the 25 ST19 isolates showed adhesion rates ranging from 0.15‰ to 11.67‰ (median: 0.92‰), while the 29 ST11 isolates demonstrated values between 0.05‰ and 24.20‰ (median: 2.54‰). Statistical comparisons using the Wilcoxon rank-sum test indicated that ST34 displayed significantly stronger adherent capacity than both ST11 ( P < 0.05) and ST19 ( P < 0.05). However, no statistically significant difference was observed between ST11 and ST19 ( P = 0.2).

Similarly, invasion assays revealed that ST34 isolates possessed invasion rates ranging from 0.07‰ to 4.67‰ (median: 0.74‰) (**Figures 2C, D**). ST19 isolates varied from 0.04‰ to 3.00‰ (median: 0.42‰), and ST11 isolates ranged from 0.02‰ to 3.00‰ (median: 0.25‰). Subsequent statistical analysis showed that ST34 had a significantly higher invasion rate than ST11 ( P < 0.05). By contrast, the differences between ST34 and ST19 did not reach statistical significance ( P = 0.066), and no significant difference was found between ST19 and ST11 ( P = 0.24).

### Comparative genomics reveals divergent virulence and resistance gene profiles among ST34, ST11 and ST19

**Figure 3:**
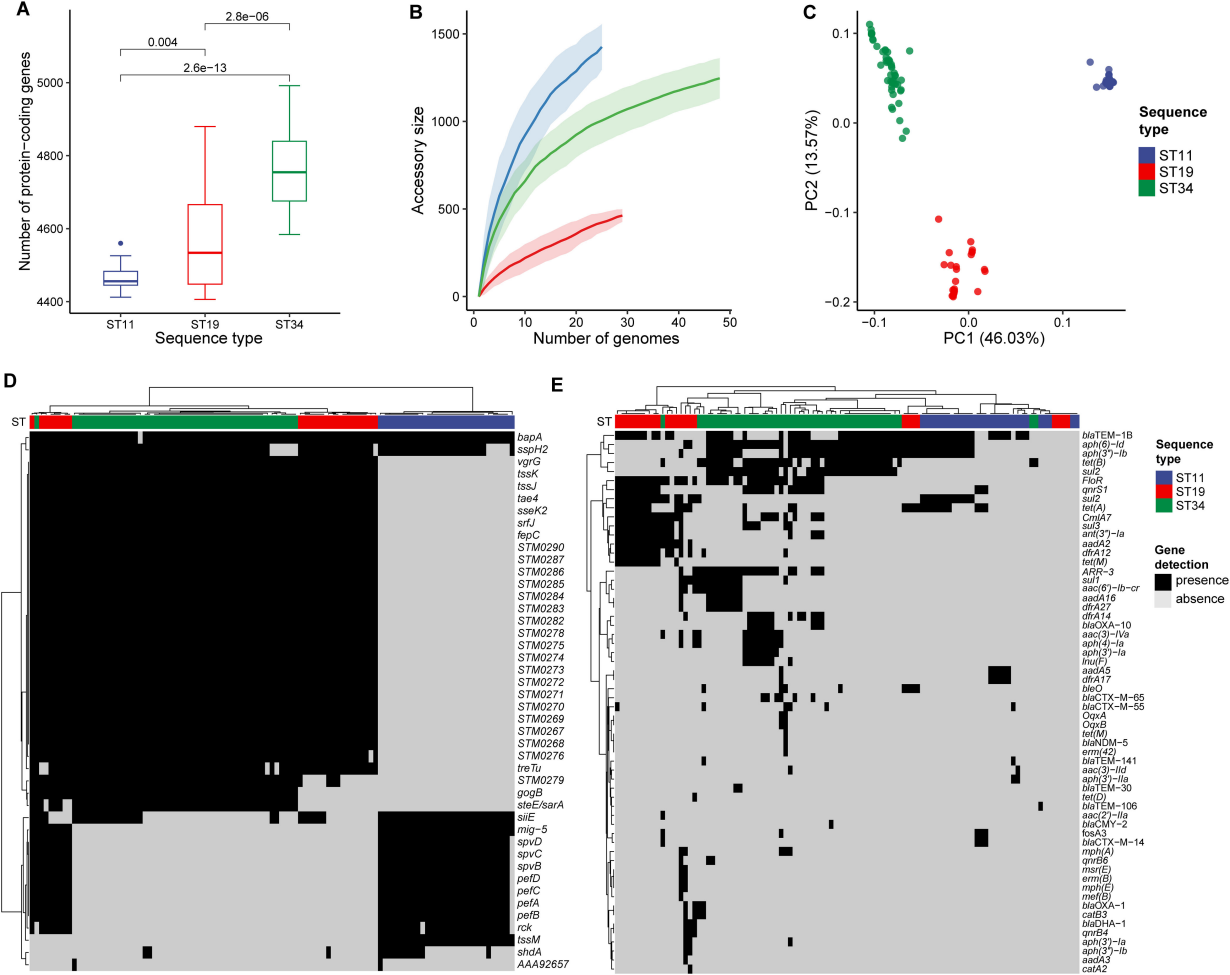
Pangenome, AMR and VF profile among ST11, ST19 and ST34. **(A)** Boxplot showing the distribution of protein-coding gene counts in the genomes of three STs. Differences in gene numbers between STs were assessed using the Wilcoxon rank-sum test. **(B)** Growth curves of the accessory genomes across the three STs. **(C)** Two-dimensional PCA visualizing pangenome diversity among the three STs. **(D)** Clustered heatmap based on the presence-absence matrix of variable VF genes (those present in <95% of strains). **(E)** Clustered heatmap generated from the presence-absence matrix of variable AMR genes (detected in <95% of strains). All panels use consistent color coding: blue for ST11, red for ST19, and green for ST34.

Genome annotation revealed significant differences in the number of protein-coding genes among the three dominant STs (Wilcoxon rank-sum test, P < 0.05), with ST34 containing the highest median number ( n = 4755), followed by ST19 ( n = 4534), and ST11 the fewest ( n = 4456) (**Figure 3A**). Comparative pangenome analysis demonstrated an open pangenome architecture for all three STs (**Figure 3B**). PCA based on gene presence-absence matrices clearly separated ST11, ST19, and ST34 genomes, consistent with their distinct evolutionary lineages derived from core-genome SNP phylogeny (**Figure 3C**).

VF profiling using the VFDB database showed that ST34 carried the most VF genes ( n = 246), followed by ST19 ( n = 177) and ST11 ( n = 149). A total of 146 VF genes were shared among all three STs, representing a conserved core virulence apparatus. Analysis of the variable VF genes, however, revealed starkly different accessory virulence strategies (**Figure 3D**). ST34 and ST19 were significantly enriched with genes encoding the Type VI Secretion System (T6SS) components (e.g., *vgrG*, *tssK*, *tssJ*) and a large cluster of genomic island/prophage-associated genes (STM gene cluster), suggesting a shared potential for inter-bacterial competition and host interaction. In contrast, ST11 was uniquely characterized by a high prevalence of pSLT-like plasmid-encoded virulence factors, including the *spv* operons (*spvBCD*) involved in intracellular proliferation, the *pef* fimbrial operons (*pefABCD*) for adhesion, and factors for serum resistance and macrophage adaptation such as *rck* and *mig-5*.

Analysis of acquired AMR genes using the ResFinder database revealed that ST34 harbored the broadest resistance gene repertoire among the three dominant STs, with 48 distinct AMR gene entries, followed by ST19 with 39 and ST11 with 16 (**Figure 3E**). Frequent ST34-associated resistance determinants included *tet(B)* and *sul2*, which appeared at high frequencies (>75%) only within this lineage. Clinically important beta-lactamase genes, including *bla*_CTX-M-55_, *bla*_CTX-M-65_, *bla*_CMY-2_, and the carbapenemase gene *bla*_NDM-5_, were detected in subsets of isolates. Notably, *bla*_NDM-5_ carriage was sporadic and consistent with the low phenotypic prevalence of carbapenem resistance. In contrast, ST11 and ST19 exhibited a more conserved set of resistance determinants with few lineage-specific markers, such as *mef(B)* conferring for macrolide in ST19 and the beta-lactamase gene *bla*_TEM-106_ in ST11.

### Transcriptomic profiling reveals distinct gene expression patterns among ST34, ST11 and ST19

To further elucidate the transcriptional landscapes of ST11, ST19, and ST34, we selected three representative isolates from each sequence type that were evenly distributed across the phylogenetic tree (**Figure 1**; **Supplementary Table 5**). These isolates were subjected to RNA sequencing, followed by transcriptome assembly and quantification. The three ST34 isolates were selected to represent phylogenetic diversity rather than the full ST34 adhesion range. The genome mapping rates ranged from 94.71% to 99.11%, and the uniquely mapped read ratios ranged from 94.41% to 98.73%. The estimated rRNA contamination rates were low, ranging from 0.178% to 0.447%. Sequencing saturation and gene-body coverage distribution analyses further indicated sufficient sequencing depth and no obvious gene-body coverage bias (**Supplementary Figure 1**; **Supplementary Table 5**).

**Figure 4:**
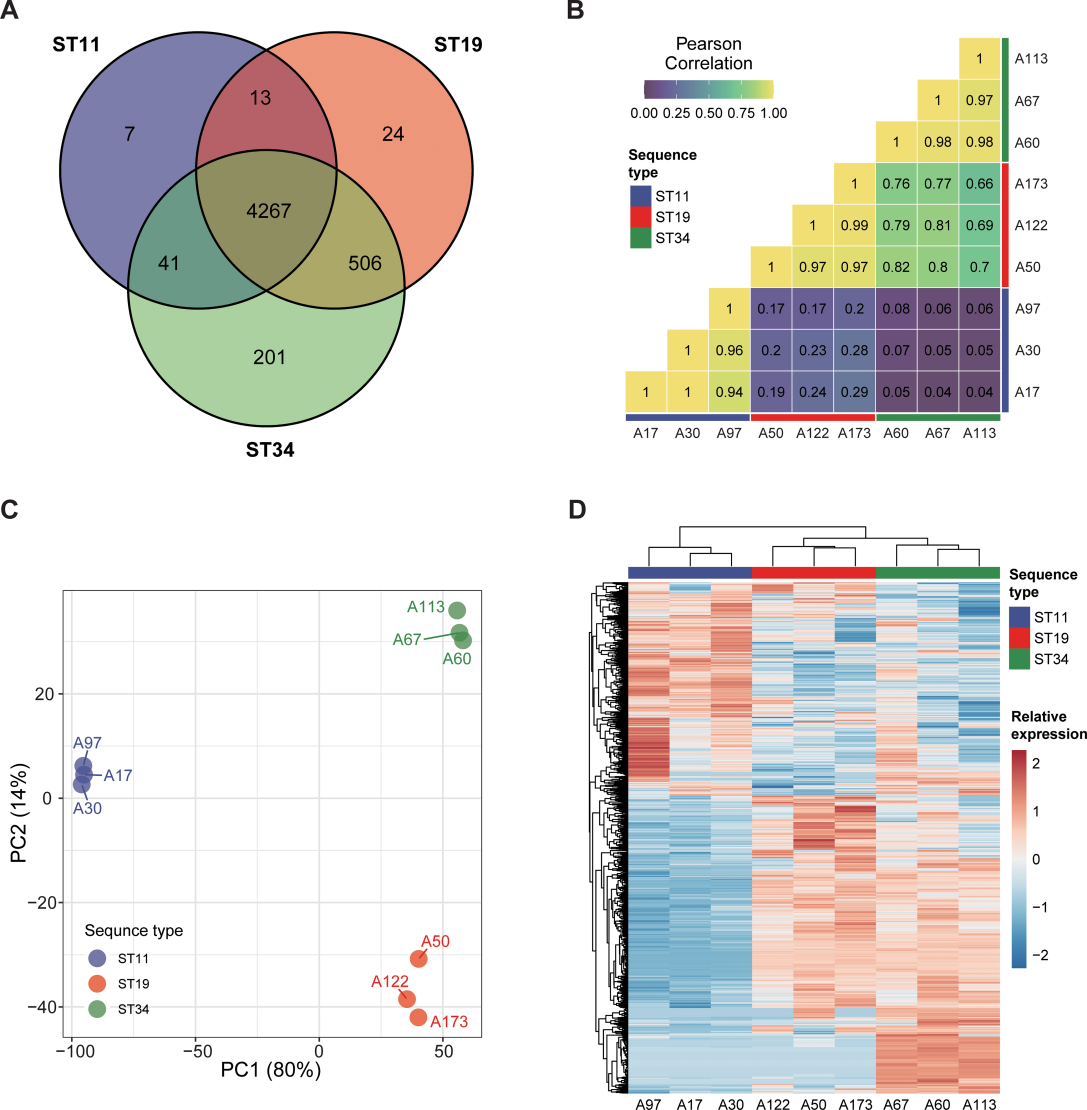
Gene expression profile among ST34, ST11 and ST19. **(A)** Venn diagram depicting the transcripts identified from transcriptome sequencing of three strains from each of the three ST types. **(B)** Pearson correlation heatmap illustrating gene expression patterns among the samples. **(C)** PCA two-dimensional scatter plot of gene expression across all samples. **(D)** Hierarchical clustering heatmap of differentially expressed genes among the three ST types. The horizontal axis of the heatmap represents individual differentially expressed genes, while the vertical axis corresponds to the samples. Heatmap color intensity reflects expression levels normalized by Z−score transformation.

A total of 5,059 transcripts were identified, among which 4,267 (84.3%) were shared across all three STs and constituted the core transcriptome (**Figure 4A**). ST34 encoded the most strain-specific transcripts ( n=201), followed by ST19 ( n=24) and ST11 ( n=7). Pairwise Pearson correlation coefficients between biological replicates exceeded 0.94 within each ST, confirming high reproducibility (**Figure 4B**). PCA based on the core transcripts revealed clear separation between ST11 and both ST19 and ST34, indicating distinct gene expression profiles among these strains (**Figure 4C**).

We identified 439 significantly upregulated and 418 significantly downregulated genes in ST19 compared to ST11 ( FDR < 0.05 and $|\log_{2}FC|>\;1$). Between ST34 and ST11, 629 genes were significantly upregulated and 370 were significantly downregulated. Notably, far fewer differentially expressed genes were observed between ST34 and ST19, with only 174 upregulated and 42 downregulated genes. Expression pattern clustering analysis of differentially expressed genes revealed that ST19 and ST34 clustered together, while ST11 formed a separate branch. This indicates that the gene expression profiles of ST19 and ST34 are more similar to each other, whereas ST11 exhibits a distinct pattern (**Figure 4D**).

### A Gifsy-1 prophage-encoded candidate virulence module is associated with the enhanced adhesion phenotype of ST34

**Figure 5:**
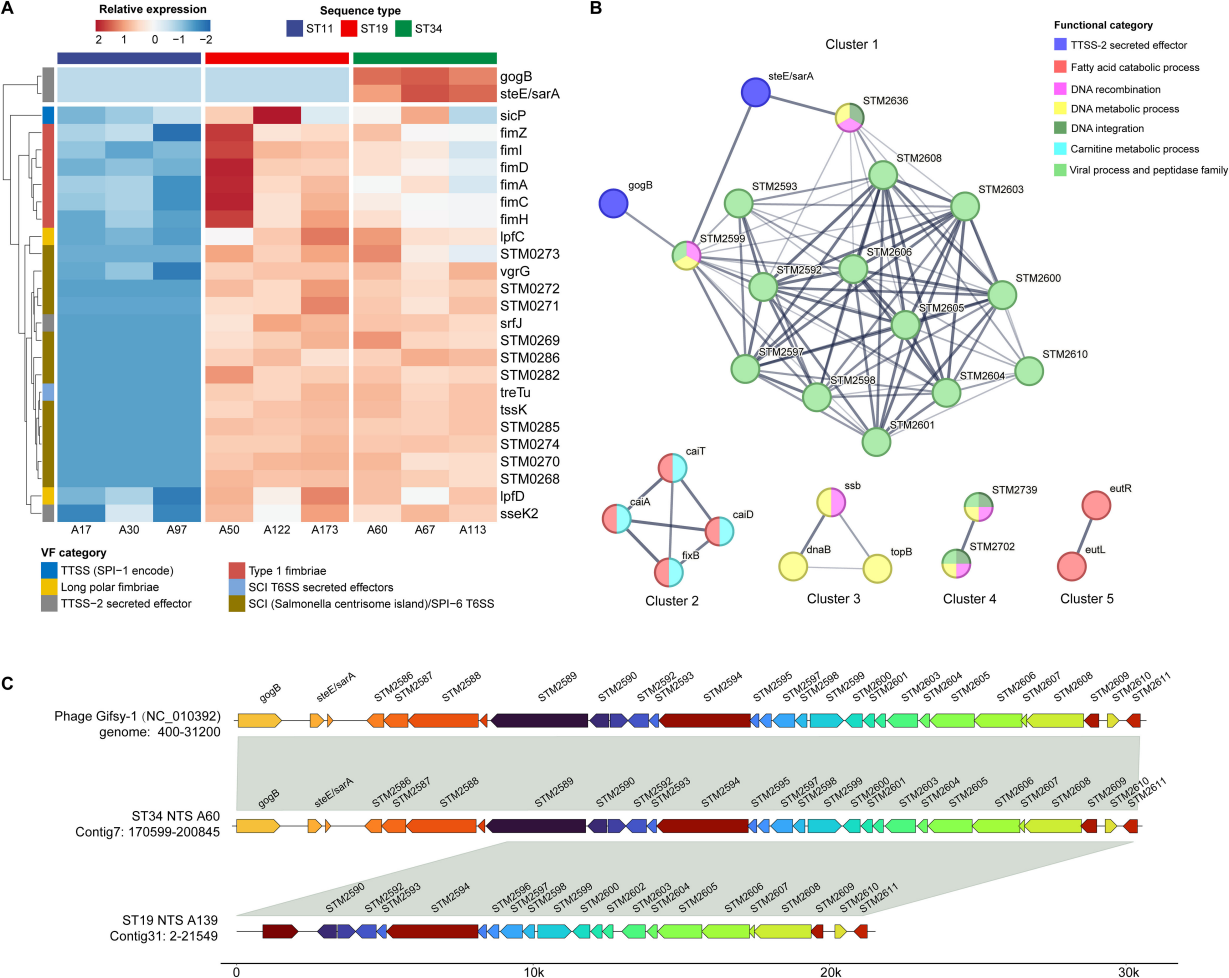
Transcriptional and Network Analyses Identify Key Virulence Factors in ST34. **(A)** Hierarchical clustering heatmap depicting the shared significantly upregulated virulence factor genes in ST34 compared to both ST11 and ST19. The horizontal axis represents individual virulence factor genes, colored on the left side according to their functional categories. The vertical axis corresponds to bacterial strains, annotated with colors at the top indicating their respective ST types. Heatmap color intensity denotes relative gene expression changes across samples. **(B)** PPI network of proteins encoded by genes that are significantly upregulated in ST34 compared to both ST11 and ST19. Nodes represent proteins, and edges indicate STRING-predicted or previously reported functional associations. Node colors correspond to functional categories enriched in the STRING database based on GO Biological Process (GO BP) and local network clustering analyses. Edge thickness reflects the confidence score of each interaction, with thicker lines indicating higher confidence. **(C)** Genomic comparison of prophage loci from strains ST19 NTS A139 and ST34 NTS A60, alongside a phage Gifsy-1 genome (RefSeq: NC_010392). Genes are shown as arrows indicating transcription direction and colored by orthologous gene families. Gray ribbons connect regions of nucleotide sequence similarity.

To elucidate the genomic feature associated with the enhanced adhesion and invasion phenotype of ST34, we interrogated the differentially expressed gene sets against the VFDB. This analysis revealed a significant enrichment of virulence-associated genes upregulated in ST34 compared to ST11, including multiple fimbrial operons (type 1 fimbriae: *fimAICDHZ*; long polar fimbriae: *lpfCD*), SPI-2 T3SS effectors (*gogB*, *steE*/*sarA*, *sseK2*, *srfJ*), T6SS components (STM cluster, *tssK*, *vgrG*, *treTu*), as well as the SPI-1 component *sicP* (**Figure 5A**). Strikingly, the comparison between ST34 and its transcriptionally similar counterpart ST19 identified a focused set of virulence-associated genes centered on *gogB* and *steE*/*sarA*. These genes were detected as ST34-associated expressed genes located within the intact Gifsy-1 prophage, suggesting that their presence and expression may contribute to the enhanced epithelial adhesion phenotype observed in ST34. Because all reads were mapped to the ST34 reference genome A60, expression patterns involving accessory or prophage genes were interpreted together with gene presence/absence data rather than as simple transcriptional downregulation events in ST11 or ST19.

To further investigate the functional relationships among the genes consistently upregulated in ST34, we performed PPI network analysis using the STRING database on the 125 overlapping genes identified from both comparisons (ST34 vs. ST11 and ST34 vs. ST19). The resulting network was clustered into five distinct functional modules (**Figure 5B**). The largest and most prominent cluster (Cluster 1) comprised 16 members, with the two key VFs GogB and SteE/SarA positioned at the topological periphery of this subnetwork. These two effectors were positioned within a functional association network that also included 14 Gifsy-1 prophage-encoded proteins, which were primarily associated with viral processes and possessed peptidase family S49 domains. This structural organization suggests that GogB and SteE/SarA, while positioned at the topological periphery of this subnetwork, may be functionally associated with this prophage-derived module, although direct regulatory or physical interactions remain to be experimentally validated.

To validate the genomic context of the ST34−specific virulence determinants identified in transcriptomic and PPI analyses, we scanned all 188 sequenced genomes for prophage regions using PHASTEST. Remarkably, all 49 ST34 isolates carried an intact Gifsy−1 prophage region (mean length=30.2kb, mean coverage ;= ;96.4%) that included *gogB*, *steE*/*sarA*, and most of the STM gene cluster identified in our network analysis (**Supplementary Table 4**). We further screened the complete genomes of 200 ST34 NTS strains collected from the BV-BRC database for the presence of the Gifsy−1 prophage region. The prophage was detected in 185 of the isolates, corresponding to a prevalence of 92.5% (**Supplementary Table 4**).

In contrast, only five ST19 isolates harbored a partial Gifsy−1 prophage region (mean length=21.5kb, mean coverage ;= ;70.0%), and no ST11 isolates contained this prophage (**Supplementary Table 4**). Alignment of representative prophage regions from one ST34 and one ST19 isolate against the reference Gifsy−1 genome confirmed that the ST34 prophage was nearly identical to the reference (99.9% nucleotide identity, 100% coverage), whereas the ST19 region was substantially truncated, with precise deletion of the *gogB* and *steE*/*sarA* genes (**Figure 5C**). Together, these results indicate that a candidate virulence-associated gene module in ST34 is located within an intact Gifsy-1 prophage, whereas this region is absent or truncated in the comparator STs.

The remaining clusters were smaller and functionally distinct: Cluster 2 ( n=4) showed enrichment for fatty acid catabolic processes and carnitine metabolism; both Cluster 3 ( n=3) and Cluster 4 ( n=2) were associated with DNA metabolic processes; Cluster 4 additionally exhibited enrichment in DNA recombination and integration; Cluster 5 ( n=2) was similarly linked to fatty acid catabolism, sharing this functional association with Cluster 2 (**Figure 5B**). This network architecture reveals that the ST34-specific transcriptional signature is dominated by a prophage-associated module centered around *GogB* and *SteE*/*SarA*, alongside distinct metabolic adaptations that may contribute to the enhanced adhesion-associated phenotype of ST34.

## Discussion

In this study, we conducted a comprehensive 7.5-year surveillance of NTS isolates from patients with acute diarrheal illness in Huzhou, China, revealing the striking predominance of three sequence types: ST34, ST11, and ST19. Through integrated analyses combining comparative genomics, quantitative cell-based infection assays, and transcriptomic profiling, we show that ST34 exhibits significantly enhanced epithelial adhesion compared with ST11 and ST19, with increased invasion observed only in comparison with ST11. Comparative genomic and transcriptomic analyses identified an intact Gifsy-1 prophage region encoding the T3SS effectors GogB and SteE/SarA as a ST34-specific candidate virulence-associated module. These findings identify a prophage-encoded candidate virulence module associated with the enhanced epithelial adhesion phenotype of the emerging ST34 clone and provide insights into the divergent pathogenic strategies employed by dominant NTS lineages.

### ST34 as an emerging dominant clone with enhanced pathogenic potential

The epidemiological shift toward ST34 predominance observed in our study aligns with global expansion of monophasic *S. Typhimurium* ST34, which has surpassed the previously dominant ST19 in multiple regions worldwide (Wu et al., 2023, 2025). In China, nationwide surveillance has identified *Salmonella* 4,[5],12:i:- (predominantly ST34) as the second most prevalent serotype among human-derived NTS isolates, following ST11 and preceding ST19 (Wang et al., 2023b). The consistent detection of ST34 across all sampling years (2017–2024) in our study suggests its stable establishment in the local population.

Beyond its epidemiological success, our functional assays indicate that ST34 exhibits enhanced epithelial adhesion, with more limited evidence for increased invasion. The median adhesion rate of ST34 (7.91‰) was approximately 8.6-fold higher than ST19 (0.92‰) and 3.1-fold higher than ST11 (2.54‰). This phenotypic advantage may contribute to clinical success, as enhanced intestinal epithelial colonization represents a critical first step in establishing infection (Chong et al., 2021).

### The Gifsy-1 prophage as a candidate module associated with enhanced adhesion in ST34

A central finding of our study is the identification of an intact Gifsy-1 prophage region as the candidate genetic module associated with ST34’s enhanced pathogenic phenotype. While prophages have long been recognized as vehicles for virulence gene transfer in *Salmonella*, the specific contribution of Gifsy-1-encoded effectors to differential pathogenicity among clinically dominant STs has not been previously demonstrated (Yates et al., 2024). Our genomic analysis revealed that all 49 ST34 isolates carried an intact Gifsy-1 prophage (mean coverage 96.4%), whereas only a minority of ST19 isolates harbored a truncated version, and ST11 isolates completely lacked this element.

Two Gifsy-1-encoded effectors—GogB and SteE/SarA—emerged as the only virulence determinants consistently upregulated in ST34 compared to both ST11 and ST19. GogB is a T3SS effector that limits inflammation by suppressing NF-κB activation, potentially allowing ST34 to establish infection with reduced immune-mediated clearance (Pilar et al., 2012). SteE/SarA promotes bacterial survival by activating the STAT3 signaling pathway and reprogramming host cell cytokine production (Gaggioli et al., 2025). The coordinated action of these effectors may create an optimal niche for bacterial colonization uniquely accessible to ST34. Notably, the clinical relevance of prophage-encoded *gogB* is further supported by a recent pangenomic analysis of a major *S. Enteritidis* outbreak, which similarly linked a Gifsy-1-like prophage carrying *gogB* to enhanced pathogenic potential (Svahn et al., 2023).

The STRING-based functional association network provides a useful framework for interpreting the ST34-associated prophage module. GogB and SteE/SarA were positioned near a cluster of 14 prophage-associated proteins, suggesting that these effectors are part of a broader Gifsy-1-associated module. However, this analysis does not demonstrate direct physical interactions, regulatory relationships, or causal effects. Therefore, we interpret the network as supportive contextual evidence for a candidate prophage-associated module rather than as proof of a coordinated regulatory mechanism.

### Divergent pathogenic and resistant strategies among dominant NTS lineages

Our comparative approach revealed that ST11, ST19, and ST34 have evolved distinct accessory virulence repertoires. ST11 was uniquely characterized by pSLT-like plasmid-encoded factors, including the *spv* operon for intracellular survival and *pef* fimbrial operon for adhesion (Guiney and Fierer, 2011). The presence of these plasmid-borne factors suggests ST11 employs a strategy centered on intracellular proliferation rather than enhanced epithelial attachment, consistent with its association with invasive infections (Wang et al., 2023b).

ST19 and ST34 shared enrichment for T6SS components and prophage-associated genes, indicating common evolutionary background. However, intact Gifsy-1 in ST34 versus truncation in ST19 represents a key differential feature associated with enhanced adhesion. The coexistence of multiple pathogenic strategies—ST34 optimized for colonization, ST11 equipped for systemic spread—may enable the population to exploit diverse transmission niches and host susceptibilities (Wang et al., 2023b).

The convergence of enhanced adhesion-associated features with extensive antimicrobial resistance in ST34 has important implications. Consistent with the ResFinder-based analysis, ST34 harbored a broader acquired AMR gene repertoire than the other dominant STs. This repertoire included clinically important resistance determinants, including ESBL or beta-lactamase genes such as *bla*_CTX-M-55_, *bla*_CTX-M-65_, and *bla*_CMY-2_, as well as sporadic detection of the carbapenemase gene *bla*_NDM-5_. Although *bla*_NDM-5_ was detected only sporadically among ST34 isolates and should not be considered a lineage-wide hallmark, its presence remains concerning because carbapenem resistance in Salmonella is uncommon but has increasingly been reported in China (Sun et al., 2025). Equally concerning is the co-carriage of ESBL genes, which confer resistance to extended-spectrum cephalosporins—a critical treatment option for invasive *Salmonella* infections, especially in children. The combination of antimicrobial resistance and a Gifsy-1-associated candidate virulence-associated module creates a pathogen profile that warrants continued surveillance.

### Limitations and future directions

Several limitations should be acknowledged. First, our functional assessments were limited to *in vitro* adhesion and invasion assays using IEC-6 cells, which cannot fully recapitulate human intestinal infection; future studies using human intestinal organoids or animal models are needed to validate *in vivo* relevance. Second, although genomic and transcriptomic analyses identified *gogB* and *steE*/*sarA* within an intact ST34-associated Gifsy-1 prophage, these data remain correlative. Causality will require genetic validation, such as isogenic deletion of *gogB*, *steE*/*sarA*, or the entire Gifsy-1 region, followed by phenotypic testing and complementation. In addition, RNA-seq was performed on three representative isolates per ST and mapped to a single ST34 reference genome, which may introduce reference bias for accessory genes and divergent prophage regions; RT-qPCR validation of *gogB* and *steE*/*sarA* was not performed. We therefore interpret this prophage region as a candidate module that may contribute to the enhanced adhesion phenotype rather than as a proven causal determinant. Third, our study focused on three dominant STs, whereas NTS populations include additional sequence types with potentially distinct pathogenic properties.

## Conclusions

This study shows that ST34 has emerged as a dominant NTS lineage with significantly enhanced epithelial adhesion compared with co-circulating ST11 and ST19 clones, whereas its invasion advantage was significant only relative to ST11. Through integrated analyses, we identified an intact Gifsy-1 prophage encoding GogB and SteE/SarA as an ST34-specific genetic feature and candidate virulence-associated module that distinguishes this lineage from co-circulating ST11 and ST19 clones. The convergence of prophage-encoded pathogenic potential with extensive antimicrobial resistance positions ST34 as a dual-threat pathogen requiring enhanced surveillance. These findings advance our understanding of how mobile genetic elements shape lineage-specific virulence in *Salmonella* and provide a molecular basis for identifying lineages with enhanced adhesion-associated genetic features within routine surveillance programs.

## Data Availability

The datasets generated in this study are available in the NCBI repository under BioProject accession number PRJNA1057448. The genome assembly is available in the GenBank database, and the RNA-Seq raw data are available in the Sequence Read Archive (SRA).

## References

[B1] Achtman M. Wain J. Weill F.-X. Nair S. Zhou Z. Sangal V. . (2012). Multilocus sequence typing as a replacement for serotyping in Salmonella enterica. PLoS Pathog. 8, e1002776. doi: 10.1371/journal.ppat.1002776 22737074 PMC3380943

[B2] Chaussé A.-M. Roche S. M. Moroldo M. Hennequet-Antier C. Holbert S. Kempf F. . (2023). Epithelial cell invasion by Salmonella Typhimurium induces modulation of genes controlled by aryl hydrocarbon receptor signaling and involved in extracellular matrix biogenesis. Virulence 14, 2158663. doi: 10.1080/21505594.2022.2158663 36600181 PMC9828750

[B3] Chen S. Zhou Y. Chen Y. Gu J. (2018). fastp: an ultra-fast all-in-one FASTQ preprocessor. Bioinformatics 34, i884–i890. doi: 10.1093/bioinformatics/bty560 30423086 PMC6129281

[B4] Chong A. Cooper K. G. Kari L. Nilsson O. R. Hillman C. Fleming B. A. . (2021). Cytosolic replication in epithelial cells fuels intestinal expansion and chronic fecal shedding of Salmonella Typhimurium. Cell. Host Microbe 29, 1177–1185. doi: 10.1016/j.chom.2021.04.017 34043959 PMC8282707

[B5] Croucher N. J. Page A. J. Connor T. R. Delaney A. J. Keane J. A. Bentley S. D. . (2015). Rapid phylogenetic analysis of large samples of recombinant bacterial whole genome sequences using Gubbins. Nucleic Acids Res. 43, e15–e15. doi: 10.1093/nar/gku1196 25414349 PMC4330336

[B6] Ferrés I. Iraola G. (2021). Protocol for post-processing of bacterial pangenome data using Pagoo pipeline. STAR Protoc. 2, 100802. doi: 10.1016/j.xpro.2021.100802 34632414 PMC8487088

[B7] Florensa A. F. Kaas R. S. Clausen P. T. L. C. Aytan-Aktug D. Aarestrup F. M. (2022). ResFinder–an open online resource for identification of antimicrobial resistance genes in next-generation sequencing data and prediction of phenotypes from genotypes. Microb. Genomics 8. doi: 10.1099/mgen.0.000748 35072601 PMC8914360

[B8] Gaggioli M. R. Jones A. G. Panagi I. Washington E. J. Loney R. E. Muench J. H. . (2025). A single amino acid in the Salmonella effector SarA/SteE triggers supraphysiological activation of STAT3 for anti-inflammatory gene expression. Cell Rep. 44, 115530. doi: 10.1016/j.celrep.2025.115530 40188438 PMC12014907

[B9] Gilchrist J. J. MacLennan C. A. Hill A. V. (2015). Genetic susceptibility to invasive Salmonella disease. Nat. Rev. Immunol. 15, 452–463. doi: 10.1038/nri3858 26109132

[B10] Guiney D. G. Fierer J. (2011). The role of the spv genes in salmonella pathogenesis. Front. Microbiol. 2. doi: 10.3389/fmicb.2011.00129 PMC3117207 21716657

[B11] Jolley K. A. Bray J. E. Maiden M. C. (2018). Open-access bacterial population genomics: BIGSdb software, the PubMLST. org website and their applications. Wellcome Open Res. 3. doi: 10.12688/wellcomeopenres.14826.1 PMC6192448 30345391

[B12] Kim D. Paggi J. M. Park C. Bennett C. Salzberg S. L. (2019). Graph-based genome alignment and genotyping with HISAT2 and HISAT-genotype. Nat. Biotechnol. 37, 907–915. doi: 10.1038/s41587-019-0201-4 31375807 PMC7605509

[B13] Kumar G. Kumar S. Jangid H. Dutta J. Shidiki A. (2025). The rise of non-typhoidal Salmonella: an emerging global public health concern. Front. Microbiol. 16, 1524287. doi: 10.3389/fmicb.2025.1524287 39967739 PMC11832534

[B14] Letunic I. Bork P. (2021). Interactive Tree Of Life (iTOL) v5: an online tool for phylogenetic tree display and annotation. Nucleic Acids Res. 49, W293–W296. doi: 10.1093/nar/gkab301 33885785 PMC8265157

[B15] Liu B. Zheng D. Zhou S. Chen L. Yang J. (2022). VFDB 2022: a general classification scheme for bacterial virulence factors. Nucleic Acids Res. 50, D912–D917. doi: 10.1093/nar/gkab1107 34850947 PMC8728188

[B16] Liu X. Wang C. Gai W. Sun Z. Fang L. Hua Z. (2024). Critical role of msgA in invasive capacity and intracellular survivability of Salmonella. Appl. Environ. Microbiol. 90, e00201–e00224. doi: 10.1128/aem.00201-24 39136487 PMC11409701

[B17] Love M. I. Huber W. Anders S. (2014). Moderated estimation of fold change and dispersion for RNA-seq data with DESeq2. Genome Biol. 15, 1–21. doi: 10.1186/s13059-014-0550-8 25516281 PMC4302049

[B18] Majowicz S. E. Musto J. Scallan E. Angulo F. J. Kirk M. O’Brien S. J. . (2010). The global burden of nontyphoidal salmonella gastroenteritis. Clin. Infect. Dis. 50, 882–889. doi: 10.1086/650733 20158401

[B19] Mezal E. H. Sabol A. Khan M. A. Ali N. Stefanova R. Khan A. A. (2014). Isolation and molecular characterization of Salmonella enterica serovar Enteritidis from poultry house and clinical samples during 2010. Food Microbiol. 38, 67–74. doi: 10.1016/j.ijfoodmicro.2013.03.021 24290628

[B20] Nguyen L.-T. Schmidt H. A. von Haeseler A. Minh B. Q. (2015). IQ-TREE: A fast and effective stochastic algorithm for estimating maximum-likelihood phylogenies. Mol. Biol. Evol. 32, 268–274. doi: 10.1093/molbev/msu300 25371430 PMC4271533

[B21] Pardos de la Gandara M. Fournet N. Bonifait L. Lefèvre S. Chemaly M. Grastilleur C. . (2023). Countrywide multi-serotype outbreak of Salmonella Bovismorbificans ST142 and monophasic Salmonella Typhimurium ST34 associated with dried pork sausages in France, September 2020* to January 2021. Eurosurveillance 28, 2200123. doi: 10.2807/1560-7917.es.2023.28.2.2200123 36695482 PMC9837855

[B22] Pertea M. Pertea G. M. Antonescu C. M. Chang T.-C. Mendell J. T. Salzberg S. L. (2015). StringTie enables improved reconstruction of a transcriptome from RNA-seq reads. Nat. Biotechnol. 33, 290–295. doi: 10.1038/nbt.3122 25690850 PMC4643835

[B23] Pilar A. V. C. Reid-Yu S. A. Cooper C. A. Mulder D. T. Coombes B. K. (2012). GogB Is an Anti-Inflammatory Effector that Limits Tissue Damage during Salmonella Infection through Interaction with Human FBXO22 and Skp1. PLoS Pathog. 8, e1002773. doi: 10.1371/journal.ppat.1002773 22761574 PMC3386239

[B24] Schwengers O. Jelonek L. Dieckmann M. A. Beyvers S. Blom J. Goesmann A. (2021). Bakta: rapid and standardized annotation of bacterial genomes via alignment-free sequence identification. Microb. Genomics 7, 685. doi: 10.1099/mgen.0.000685 34739369 PMC8743544

[B25] Snipen L. Liland K. H. (2015). micropan: an R-package for microbial pan-genomics. BMC Bioinf. 16, 79. doi: 10.1186/s12859-015-0517-0 25888166 PMC4375852

[B26] Sun H. Cui Z. Du X. Fan F. Sun B. Diao B. . (2025). Prevalence of Salmonella enterica Serotype 4,[5], 12: i:-from Human Sources: Antimicrobial Resistance, Genotypic Diversity and Emergence of Carbapenem Resistance—China 2017–2023. China CDC Weekly 7, 821. doi: 10.1001/jama.2013.283124 40620292 PMC12228071

[B27] Svahn A. J. Suster C. J. E. Chang S. L. Rockett R. J. Sim E. M. Cliff O. M. . (2023). Pangenome analysis of a salmonella enteritidis population links a major outbreak to a gifsy-1-like prophage containing anti-inflammatory gene gogB. Microbiol. Spectr. 11, e02791–e02722. doi: 10.1128/spectrum.02791-22 36916949 PMC10100743

[B28] Szklarczyk D. Morris J. H. Cook H. Kuhn M. Wyder S. Simonovic M. . (2017). The STRING database in 2017: quality-controlled protein–protein association networks, made broadly accessible. Nucleic Acids Res. 45, D362–D368. doi: 10.1093/nar/gkw937 27924014 PMC5210637

[B29] Tonkin-Hill G. Gladstone R. A. Pöntinen A. K. Arredondo-Alonso S. Bentley S. D. Corander J. (2023). Robust analysis of prokaryotic pangenome gene gain and loss rates with Panstripe. Genome Res. 33, 129–140. doi: 10.1101/gr.277340.122 36669850 PMC9977150

[B30] Tonkin-Hill G. MacAlasdair N. Ruis C. Weimann A. Horesh G. Lees J. A. . (2020). Producing polished prokaryotic pangenomes with the Panaroo pipeline. Genome Biol. 21, 180. doi: 10.1186/s13059-020-02090-4 32698896 PMC7376924

[B31] Treangen T. J. Ondov B. D. Koren S. Phillippy A. M. (2014). The Harvest suite for rapid core-genome alignment and visualization of thousands of intraspecific microbial genomes. Genome Biol. 15, 524. doi: 10.1186/s13059-014-0524-x 25410596 PMC4262987

[B32] Wang Y. Liu Y. Lyu N. Li Z. Ma S. Cao D. . (2023b). The temporal dynamics of antimicrobial-resistant Salmonella enterica and predominant serovars in China. Natl. Sci. Rev. 10, nwac269. doi: 10.1093/nsr/nwac269 37035020 PMC10076184

[B33] Wang S. Mirmiran S. D. Li X. Li X. Zhang F. Duan X. . (2023a). Temperate phage influence virulence and biofilm-forming of Salmonella Typhimurium and enhance the ability to contaminate food product. Int. J. Food Microbiol. 398, 110223. doi: 10.1016/j.ijfoodmicro.2023.110223 37120944

[B34] Wick R. R. Judd L. M. Gorrie C. L. Holt K. E. (2017). Unicycler: resolving bacterial genome assemblies from short and long sequencing reads. PLoS Comput. Biol. 13, e1005595. doi: 10.1371/journal.pcbi.1005595 28594827 PMC5481147

[B35] Wu X. Du J. Zhou X. Peng X. Jia C. Wang B. . (2025). Genomic epidemiology and public health implications of zoonotic monophasic Salmonella Typhimurium ST34. Front. Cell. Infect. Microbiol. 15. doi: 10.3389/fcimb.2025.1490183 PMC11933091 40134787

[B36] Wu Y. Jiang T. Bao D. Yue M. Jia H. Wu J. . (2023). Global population structure and genomic surveillance framework of carbapenem-resistant Salmonella enterica. Drug Resistance Updates 68, 100953. doi: 10.1016/j.drup.2023.100953 36841133

[B37] Yan S. Zhang W. Li C. Liu X. Zhu L. Chen L. . (2021). Serotyping, MLST, and Core genome MLST analysis of Salmonella enterica from different sources in China during 2004–2019. Front. Microbiol. 12, 688614. doi: 10.3389/fmicb.2021.688614 34603224 PMC8481815

[B38] Yates C. R. Nguyen A. Liao J. Cheng R. A. (2024). What’s on a prophage: analysis of Salmonella spp. prophages identifies a diverse range of cargo with multiple virulence-and metabolism-associated functions. Msphere 9, e00031–e00024. doi: 10.1128/msphere.00031-24 38775467 PMC11332146

[B39] Zhang S. Den Bakker H. C. Li S. Chen J. Dinsmore B. A. Lane C. . (2019). SeqSero2: rapid and improved salmonella serotype determination using whole-genome sequencing data. Appl. Environ. Microbiol. 85, e01746–e01719. doi: 10.1128/AEM.01746-19 31540993 PMC6856333

