# Supplementary material for: Genomic and transcriptomic analyses reveal a Gifsy-1 prophage-encoded virulence module associated with enhanced epithelial adhesion of Salmonella ST34 in Huzhou, China
Source: Front Cell Infect Microbiol. 2026 Jul 24;16:1849969. doi: 10.3389/fcimb.2026.1849969 (PMC13447223; doi:10.3389/fcimb.2026.1849969)
Supplement: Supplementary file 1 [file Image1.pdf]

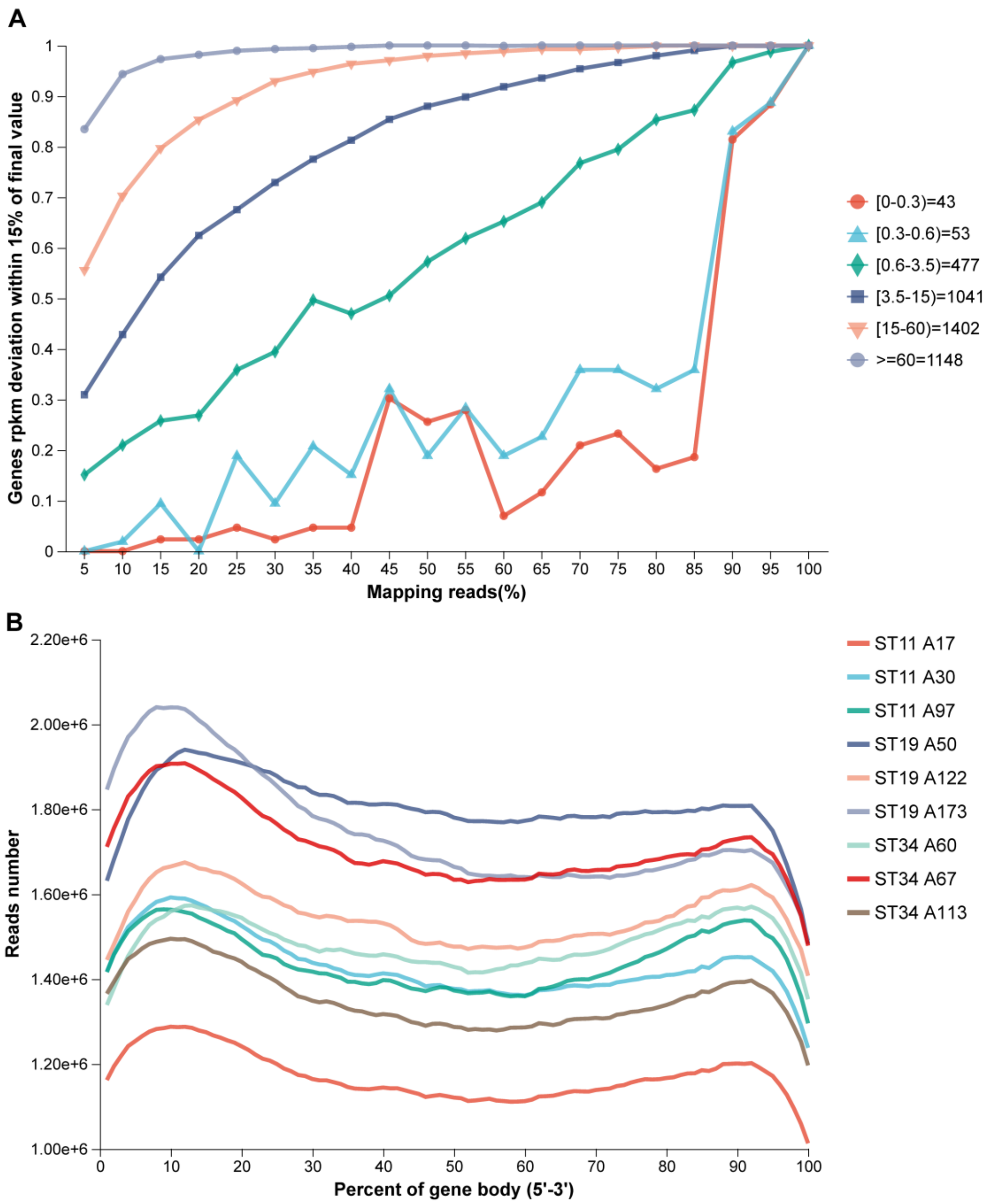

Supplementary Figure S1. RNA-seq sequencing saturation and gene-body coverage distribution. (A) Sequencing saturation curves for the nine RNA-seq samples. The x-axis indicates the percentage of effectively mapped reads randomly sampled for expression estimation, and the y-axis indicates the proportion of genes with expression estimates within 15% deviation from the final value. (B) Gene-body coverage distribution across annotated genes. The x-axis indicates the relative position along gene bodies from the 5′ end to the 3′ end, and the y-axis indicates the normalized read coverage. The absence of strong 5′ or 3′ bias indicates generally uniform gene-body coverage.
